# Formation of a heterooctameric complex between aspartate α-decarboxylase and its cognate activating factor, PanZ, is CoA-dependent

**DOI:** 10.1016/j.bbrc.2012.08.084

**Published:** 2012-09-28

**Authors:** Diana C.F. Monteiro, Michael D. Rugen, Dale Shepherd, Shingo Nozaki, Hironori Niki, Michael E. Webb

**Affiliations:** a School of Chemistry, University of Leeds, Leeds LS2 9JT, United Kingdom; b Astbury Centre for Structural Molecular Biology, University of Leeds, Leeds LS2 9JT, United Kingdom; c Microbial Genetics Laboratory, Genetic Strains Research Center, National Institute of Genetics, 1111 Yata, Mishima, Shizuoka 411-8540, Japan; d Department of Genetics, Graduate University for Advanced Studies (Sokendai), 1111 Yata, Mishima, Shizuoka 411-8540, Japan

**Keywords:** Pantothenate, Vitamin biosynthesis, Pyruvoyl-cofactor, Post-translational modification

## Abstract

The existence of a fifth essential protein for pantothenate biosynthesis in some enteric bacteria has recently been reported by Stuecker et al. [10] and Nozaki et al. (in press) [9]. This protein, PanZ, catalyses the activation of the PanD zymogen to form ADC and is essential for prototrophic growth. In this paper, we characterise the interaction of PanZ with coenzyme A and a constitutively inactive mutant of PanD using a combination of isothermal titration calorimetry and mass spectrometry. These approaches reveal that the two proteins interact with nanomolar affinity in a CoA-dependent fashion to form a heterooctameric complex.

## Highlights

► PanZ is required for formation of the pyruvoyl-cofactor in PanD (ADC) *in vivo*. ► PanZ and PanD interact with nanomolar affinity. ► Interaction of PanZ and PanD is dependent upon coenzyme A. ► PanZ and PanD form a heterooctameric complex which binds four molecules of CoA.

## 1 Introduction

**Fig. 1:**
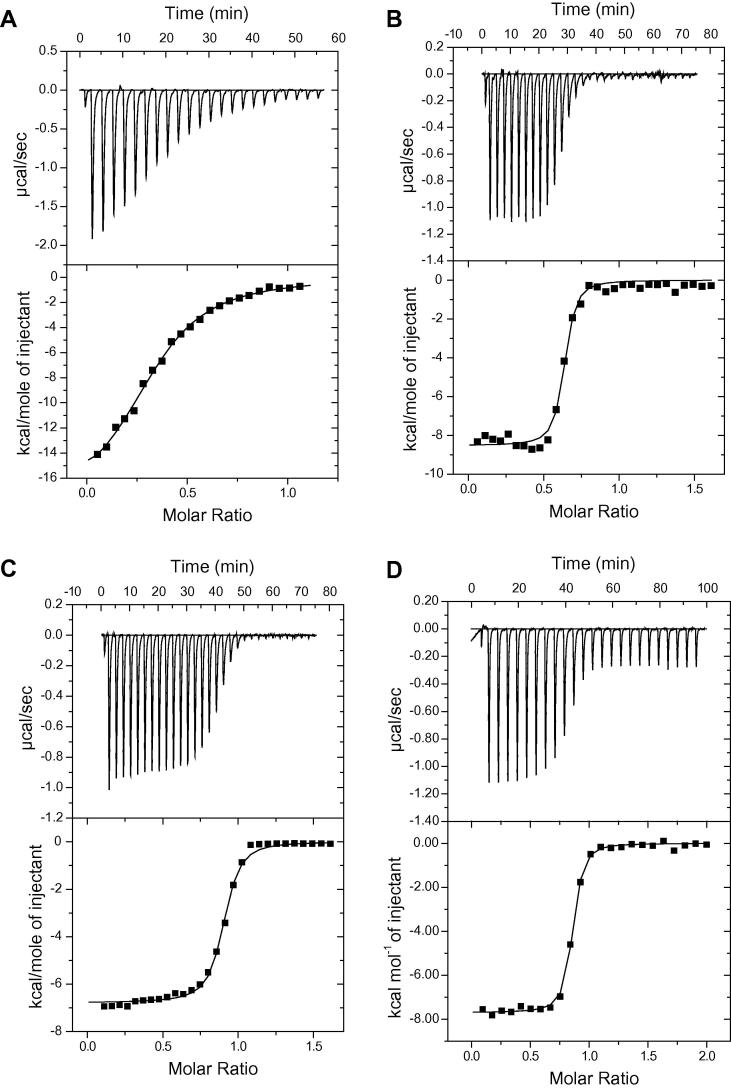
Calorimetric analysis of interaction between PanD and PanZ. (A) Representative titration of 400 μM CoA into 60 μM purified PanZ reveals a sub-stoichiometric number of available binding sites. (B) Representative titration of 394 μM PanD-T57V into 59 μM PanZ without added CoA. (C) Titration of 394 μM PanD-T57V into 59 μM PanZ after addition of 110 μM CoA to each sample. (D) Titration of 348 μM PanZ into 31 μM PanD-T57V after addition of 110 μM CoA to each sample.

Pyruvoyl-dependent enzymes [1] are central components of several biosynthetic pathways including those for the polyamines, phosphatidylethanolamine and pantothenate. The catalytic covalently-bound pyruvoyl group is formed as a result of intramolecular rearrangement of a serine residue to form an ester intermediate [2], which is cleaved to generate a dehydroalanyl residue which is hydrolysed to generate the pyruvoyl group (Fig. 1). This process was originally thought to be autocatalytic however formation of active S-adenosylmethionine decarboxylase in human polyamine biosynthesis is enhanced by putrescine, the substrate for the next enzyme in the pathway [3]. More recently, protein cofactors responsible for activation of these enzymes have been reported, such as HdcB, identified in 2011, which activates histidine decarboxylase in Lactobacillus [4].

Aspartate α-decarboxylase is responsible for the formation of β-alanine as part of the bacterial pantothenate biosynthesis pathway [5]. The enzyme was first purified and determined to be pyruvoyl-dependent by Williamson and Brown [6], it was later cloned and overexpressed by Ramjee et al. [7] who characterised its activation by mass spectrometry and by Albert et al. who determined the structure of the enzyme and identified the ester intermediate in activation [2]. Schmitzberger et al. [8] subsequently reported the structure of the unprocessed zymogen and investigated the sequence dependence of activation *in vitro*. This process is very slow and the observed rate of activation was inconsistent with the growth rate of *Escherichia* *coli* suggesting the presence of an activating factor. Such an activating factor was recently identified independently as PanZ in *E. coli* [9] and PanM in *Salmonella enterica* [10], a putative acyl transferase essential for pantothenate biosynthesis in these organisms as mutation of the gene leads to pantothenate auxotrophy. Stuecker et al. [11] have recently reported that, although acetyl CoA is required for activation, acetyl transfer to *Salmonella* PanD does not occur, and that disruption of CoA binding inhibits protein–protein interaction *in vivo*. In this manuscript, we report a complementary analysis of the protein complex between *E. coli* PanD and PanZ *in vitro* using isothermal titration calorimetry and mass spectrometry and thereby determine the stoichiometry and affinity of the components of this protein complex.

## 2 Materials and methods

### 2.1 Protein expression and purification

His-tagged ADC-T57V and PanZ were overexpressed using an *E. coli* ΔpanD ΔpanZ (DE3) cell strain [9]. Lambda (DE3) lysogens were generated using a Novagen λDE3 lysogenisation kit. His-tagged ADC-T57V and PanZ were overexpressed from the vectors pRSETA-ADC-T57V (Webb et al., unpublished results) and pCA24N(-GFP)-his-panZ using an autoinduction protocol [12]. Cells were isolated by centrifugation (6000*g*, 15 min), resuspended in lysis buffer (50 mM potassium phosphate, 300 mM NaCl, 10 mM imidazole, pH 7.4), mechanically lysed using a Constant Systems cell disrupter (20 kpsi) and the lysate cleared by centrifugation (30,000*g*, 45 min). DNase I (Roche, ∼0.5 mg/l) was added to the cleared lysate before application to a Ni-NTA agarose (Qiagen) column under gravity flow and the column washed with 10 column volumes of wash buffer (50 mM potassium phosphate, 300 mM NaCl, 50 mM imidazole, pH 7.4) before elution of protein fractions using elution buffer (50 mM potassium phosphate, 300 mM NaCl, 250 mM imidazole, pH 7.4). Combined protein-containing fractions identified by SDS–PAGE were concentrated using centrifugal concentrations (Amicon, 10 kDa MWCO) and applied to a Hi-Load 26/60 Superdex 75 size-exclusion column using an Akta Purifier FPLC system. The protein was isocratically eluted (50 mM Tris–HCl, 100 mM NaCl, 0.1 mM DTT, pH 7.4) to yield pure protein samples. Protein identity was confirmed by electrospray mass spectrometry using a Bruker HCT-Ultra LC–MS system. Protein concentrations were determined by UV absorption at 280 nm in 6 M GdmCl using theoretical estimates for the protein concentration based on the primary sequence of the protein (ProtParam, [13]: PanD-T57V *ε*_280_ 15470 M^−1^ cm^−1^, PanZ *ε*_280_ 26470 M^−1^ cm^−1^). Corrections for copurified CoA in the PanZ sample were based on *ε*_280_ 2370 M^−1^ cm^−1^.

### 2.2 Isothermal titration calorimetry

Isothermal titration calorimetry experiments were performed using a Microcal VP-ITC (GE) thermostatted at 25 °C. The ligand sample was loaded into the sample cell (∼1.5 ml) and the titrant into the sample syringe (298 μl). Each titration series consisted of a 2 μl injection followed by 29 injections of 10 μl. Samples were prepared in 50 mM Tris–HCl, 100 mM NaCl, pH 7.4, 0.1 mM DTT for titrations in the presence of additional CoA, added to both the ligand and titrant sample to the same final concentration disregarding any copurified CoA. Data were analysed in Origin 6.5. After baseline substraction, data was fitted to a single site-binding model. For analysis of CoA-binding, the final cell CoA concentration and protein concentrations were recalculated based on estimates of the CoA concentration obtained by binding of ADC-T57V.

### 2.3 Noncovalent electrospray mass spectrometry

Spectra were acquired using a Synapt HDMS orthogonal acceleration quadrupole-travelling wave-time-of-flight mass spectrometer (Micromass UK Ltd., Waters Corp., Manchester, UK). PanZ-CoA and ADC-T57V protein solutions were buffer exchanged into 100 mM ammonium acetate, pH 6.8, using Micro Bio-Spin 6 Chromatography columns (Bio-Rad Life Science, Hemel Hempstead, UK). The proteins were mixed at a 1:1 ratio (monomer concentration) to generate the complex. The samples were electrosprayed from gold/platinum-plated borosilicate capillaries fabricated in-house using a P-97 micropipette puller (Sutter Instrument Company, Novato, CA) and a sputter coater (Polaron SC7620; Quorum Technologies Ltd., Kent, UK). The electrospray capillary voltage was set at 1.7 kV and the sample cone voltage at 40–60 V. To improve the resolution of the complex signals by collisional activation, the voltage of the trap region of the spectrometer was increased to 50 V. The instrument source pressure was 3 mbar and the trap gas flow rate was 5 ml min^−1^.

## 3 Results

PanZ has recently been shown to be responsible for the activation of PanD to form catalytically-active ADC both *in vivo* and *in vitro* [9–11]. This activation proceeds via rearrangement of the peptide backbone of Ser25 to generate an ester intermediate which is cleaved to form an N-terminal dehydroalanyl residue which hydrolyses to form a pyruvoyl group (see Fig. 1). We wished to investigate the nature of the interaction between the two proteins, PanZ and PanD. Since PanZ catalyses the activation of PanD, we anticipated that any analysis of the interaction of PanZ with WT PanD would be complicated by the presence of the activation reaction, the affinity of PanZ for the activated form may be different to that for the zymogen. We therefore chose to use a constitutive inactive form of ADC in which residue Thr57 is mutated to valine (PDB: 4azd). This residue provides an essential hydrogen-bond to the carbonyl of Gly24, thereby promoting the rearrangement to form the ester intermediate [8]. We have previously demonstrated that this protein is purified as the inactive zymogen in standard *E. coli* expression strains (in which PanZ is expressed and WT protein is purified as the partially activated zymogen) suggesting that PanD-T57V is not activated by PanZ (unpublished results).

PanD-T57V and PanZ were overexpressed in *E. coli* Δ*panD* Δ*panZ* (DE3) from the vectors pRSETA-ADC-T57V and pCA24N(-GFP)-his-panZ using standard autoinduction protocols for lactose and arabinose induction respectively [12]. The double knock-out strain of *E. coli* was used to ensure that each protein preparation was not contaminated with exogenously expressed untagged PanZ or PanD: the molecular weights of the two proteins are sufficiently close that small quantities of contaminating proteins could be readily masked by the overexpressed partner. Both proteins were purified to homogeneity as determined by SDS–PAGE and size-exclusion chromatography. The proteins’ elution volumes were consistent with previous observations with being present in solution as a monomer (PanZ) and a tetramer (PanD), consistent.

**Fig. 2:**
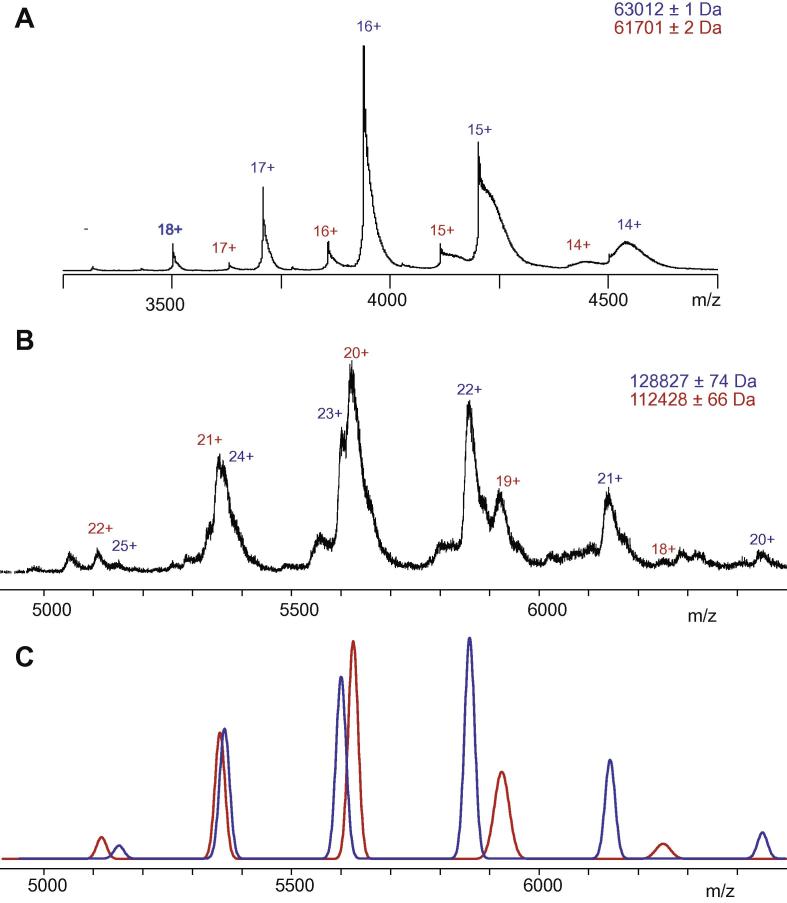
Electrospray MS analysis of PanD-T57V and its complex with PanZ-CoA. (A) ESMS spectrum of PanD-T57V tetramer. (B) ESMS spectrum of PanD-T57V in the presence of PanZ-CoA reveals a mixture of species corresponding to a tetramer of PanD-T57V bound to either 3 or 4 equivalents of PanZ-CoA. (C) Simulation of peak positions for PanD–PanZ complexes matches observed peak positions.

Cort et al. (PDB: 2k5t) noted that after overexpression in *E. coli*, PanZ is purified as a mixture of free protein and ligand-bound to coenzyme A (CoA) and the reported structure is that of the CoA-bound form. We initially investigated the binding of CoA and acetyl CoA to PanZ using ITC; titration of CoA and AcCoA into PanZ showed binding in substoichiometric ratios but with micromolar affinity in both cases (Fig. 2, Table 1). We next investigated the interaction of PanZ with the ADC-T57V site-directed mutant by titration of PanZ into PanD, for which a sub-stoichiometric interaction was again observed (*N* = 0.616). The affinity of the interaction was however tight, with a fitted dissociation constant of 58 ± 11 nM. A reverse titration in which ADC-T57V was titrated into PanZ yielded a similar value for the dissociation constant (120 ± 30 nM) but an apparent super-stoichiometric number of binding sites (*N* = 1.33).

We hypothesised that the observation of substoichiometric binding could be attributed to the interaction between the two proteins being dependent upon the presence of CoA. We therefore repeated the titration experiments in the presence of 25, 55 and 110 μM CoA. In the case of titrations of PanZ into PanD, a gradually increasing relative stoichiometry of interaction was seen in response to increasing concentrations of CoA. For the reverse titration series of PanD into PanZ a decrease in the apparent stoichiometry was observed with addition of CoA. The titration experiments with 110 μM CoA added can be simply fitted to single-site binding models to yield consistent values for the dissociation constant.

The presence of CoA in the purified samples of PanZ complicates numerical analysis of the binding data. We attempted to generate PanZ free of CoA, however protein samples precipitated upon dialysis suggesting that the CoA is required for protein stability at moderate to high concentrations. This leads to an underestimate of the affinity between CoA and PanZ since the solution of the sample cell contains an unknown number of equivalents of bound CoA. Additionally, copurified CoA in the PanZ preparation makes a small contribution to the 280 nm absorption measurements used to calculate the concentration of PanZ (denatured sample in GdmCl). We therefore made the assumption that only CoA-bound PanZ interacts with PanD, which allowed us to estimate the concentration of CoA-bound PanZ on the basis of the stoichiometry of the PanZ–PanD interaction in the absence of added CoA. This allowed the back-calculation of the true concentration of PanZ (both bound and *apo*) in the cell, in addition to an estimate for a starting concentration of CoA. Using the newly estimated PanZ concentration we refitted the binding titration for CoA yielding a new estimate for the *K*_d_ of 18 μM, 2.5-fold lower than the uncorrected value (Table 2). In this analysis the number of binding sites for CoA was fully consistent with a 1:1 binding interaction (*N* = 0.97).

The same approach was then used to correct the protein concentration estimates for other titration experiments. In the case of titrations of PanD into PanZ, the addition of 25 μM CoA to protein in both the syringe and cell is sufficient to saturate PanZ and the titration curve fits with a single-site binding model. For the reverse titration series of PanZ into PanD, PanZ only saturates at 110 μM CoA concentration – at lower concentrations some of the PanZ in the injection syringe is in the apo form, and the observed titration curve is therefore derived from both the interaction of PanZ with CoA and the interaction of PanZ-CoA with PanD (see Fig. 2). Since the enthalpy for the CoA-PanZ interaction (−18 kcal mol^−1^) is greater than that for PanZ–PanD interaction (−7 kcal mol^−1^), the former signal is dominant and it is not possible to fit these curves using a physically meaningful model. The final fitting parameters for the protein–protein interaction are shown in Table 2. The final values for the stoichiometry of interaction (*N* = 0.9) are consistent with a 1:1 binding model with mid-nanomolar affinity given the potential error in protein concentration estimates.

**Fig. 3:**
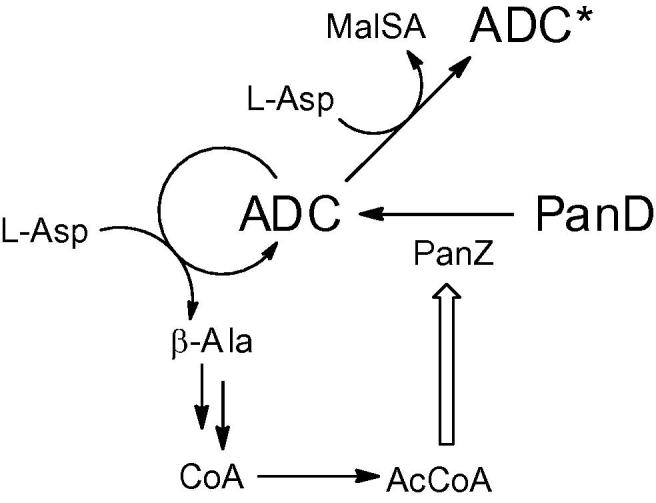
Summary of known regulatory mechanisms for PanD activation and inactivation *in vivo*. ADC is formed from PanD via action of PanZ which is stimulated by AcCoA [11]. Following activation, ADC catalyses the formation of β-alanine for CoA biosynthesis in addition to its own inactivation (to form ADC^∗^) with concomitant formation of malonic acid semialdehyde (malSA) [15].

The two series of titration experiments suggest that the two proteins and CoA are in an approximately 1:1:1 ratio. We used electrospray mass spectrometry of the proteins to determine the true stoichiometry of the interaction. We initially examined homomultimerisation of each of the proteins independently. The mass spectrum of PanZ revealed two major species (*m*/*z* 15614 and 16381 Da) corresponding to apo PanZ and PanZ bound to CoA: sufficient tertiary structure is retained in the gas-phase to maintain cofactor binding. We also observed an additional minor component at higher MW (*m*/*z* 17148 Da), resembling PanZ bound to two equivalents of CoA – we hypothesise that this second species corresponds to PanZ bound to the covalent CoA disulphide. For PanD, a major component (*m*/*z* 63.0 kDa) consistent with the tetramer of T57V together with a smaller component (*m*/*z* 61.7 kDa) corresponding to a heterotetrameric species in which C-terminal truncation had occurred in one protomer (Fig. 3b). This was confirmed by MS–MS of this species to dissociate the tetramer into full length protomer (*m*/*z* 15.753 kDa) and a truncated polypeptide (14.442 kDa).

We next investigated the mass spectrum of a mixture of PanZ and PanD in conditions of excess CoA (Fig. 3b). We observed two species, a major component with mass 128.83 ± 0.07 kDa corresponding to the unproteolysed tetramer of PanD-T57V binding to four equivalents of PanZ-CoA and a smaller component with mass 112.43 ± 0.07 kDa corresponding to the truncated tetramer of PanD-T57V bound to three equivalents of PanZ-CoA. No peaks corresponding to PanD-T57V bound to apo-PanZ were observed even when CoA concentrations were substoichiometric (not shown) and no peaks corresponding to interaction of the PanZ–CoA_2_ complex with PanD were observed. These observations confirm that the interaction of PanZ and PanD is CoA-dependent as suggested by analysis of the ITC data.

## 4 Discussion

PanZ is responsible for the activation of PanD to form catalytically active ADC *in vitro*. Using a constitutively inactive site-directed mutant of PanD, we have carried out the first biophysical characterisation of the complex formed between these two proteins *in vitro*. We demonstrate that the proteins interact directly to form a high affinity 4:4 heterooctameric complex in the presence of coenzyme A. In the absence of CoA, no complex is formed. These results agree with the mutational analysis recently reported by Stuecker et al. [11] who reported that mutations in the CoA-binding pocket of PanM from *S. enterica* lead to loss of catalytic activity accompanied by loss of protein–protein interaction as determined indirectly by yeast-two-hybrid assay. We have demonstrated that this nanomolar affinity interaction is CoA-dependent through two independent biophysical assays.

The precise manner in which this process is regulated remains unclear. Stuecker et al. [11] have demonstrated that activation of PanD is dependent upon binding of acetyl CoA but that acetylation of PanD does not occur. We have shown the physical interaction still occurs in the presence of coenzyme A. Since CoA alone has been shown to be insufficient for activation, this suggests a mechanism whereby non-covalent interaction between the acetyl group of acetyl CoA and the PanD zymogen may be required to induce activation. This suggests a mechanism for regulated activation, in that accumulation of acetyl CoA will induce production of activated PanD, thereby increasing the cellular pool of pantothenate and CoA for fatty acid biosynthesis. Activation of β-alanine supply is therefore tightly regulated in *E. coli*, previous observations of catalysis by ADC suggest that inactivation is similarly regulated. ADC is a relatively inefficient enzyme (*k*_cat_ ∼ 10 min^−1^) and the enzyme is readily auto-inactivated. During the catalytic cycle, reprotonation of the extended β-azadienolate formed as a result of decarboxylation can occur at two positions. One of those, attributed to Tyr58 by Saldanha et al. [14], regenerates the pyruvoyl group and generates β-alanine, whereas the alternative protonation leads to malonate semialdehyde and an N-terminal alanine residue in the protein (Fig. 3). As part of an evaluation of ADC as a reagent for biotechnology, Könst et al. [15] measured the rate of inactivation *in vitro*, and showed that on average it occurred after 2400 catalytic cycles. Should this be the case *in vivo*, this mechanism provides a fundamental limit on the processivity of ADC, suggesting that both the activation and inactivation of PanD is tightly regulated.

In summary, we have demonstrated that physical interaction of PanD and PanZ is dependent upon the presence of coenzyme A, which has previously been shown to be necessary but not-sufficient for acetyl CoA induced activation [11]. We have reported the stoichiometry (1:1) and affinity (∼100 nM) of this essential protein interaction and are now investigating the structural basis for both the interaction and activation of PanD to form ADC by PanZ.

## Figures and Tables

**Table 1:** Representative calculated binding data for CoA binding to PanZ.

Titrant	Ligand	Number of binding sites (*N*)	*K*_a_ × 10^5^ (M^−1^)	K_d_ (μM)	Δ*H* (kcal mol^−1^)
CoA	PanZ	0.34	1.83 ± 0.15	5.46 ± 0.45	−18.32 ± 0.55
CoA⁎	PanZ	0.97	5.13 ± 0.42	1.95 ± 0.16	−18.32 ± 0.54

⁎ After correction for concentration of bound CoA.

**Table 2:** Representative calculated binding data for interaction of PanZ-CoA with PanD-T57V.

Titrant	Ligand	Number of binding sites (*N*)	*K*_a_ × 10^6^ (M^−1^)	*K*_d_ (nM)	Δ*H* (kcal mol^−1^)
ADC	PanZ	0.62	8.41 ± 2.2	119 ± 31	−3.5 ± 0.1
ADC + 25 μM CoA	PanZ + 25 μM CoA	0.93	4.92 ± 0.40	203 ± 17	−8.67 ± 0.04
ADC + 110 μM CoA	PanZ + 110 μM CoA	0.88	4.97 ± 0.61	201 ± 25	−7.01 ± 0.05
PanZ	ADC	1.33	17.2 ± 3.4	58.1 ± 11.5	−5.22 ± 0.04
PanZ + 110 μM CoA	ADC + 110 μM CoA	0.82	17.4 ± 2.0	57.5 ± 6.6	−7.62 ± 0.04
